# Landscape Composition and Spatial Prediction of Alveolar Echinococcosis in Southern Ningxia, China

**DOI:** 10.1371/journal.pntd.0000287

**Published:** 2008-09-03

**Authors:** David R. J. Pleydell, Yu Rong Yang, F. Mark Danson, Francis Raoul, Philip S. Craig, Donald P. McManus, Dominique A. Vuitton, Qian Wang, Patrick Giraudoux

**Affiliations:** 1 Department of Chrono-environment, UMR UFC/CNRS 6249 aff. INRA, Université de Franche-Comté, Besançon, France; 2 Ningxia Medical College, Ningxia Hui Autonomous Region, People's Republic of China; 3 Queensland Institute of Medical Research, Brisbane, Queensland, Australia; 4 University of Salford, Salford, United Kingdom; 5 Université de Franche-Comté, Besançon, France; 6 Sichuan Provincial Center for Diseases Control and Prevention, Chengdu, Sichuan, People's Republic of China; London School of Hygiene & Tropical Medicine, United Kingdom

## Abstract

**Background:**

Alveolar echinococcosis (AE) presents a serious public health challenge within China. Mass screening ultrasound surveys can detect pre-symptomatic AE, but targeting areas identified from hospital records is inefficient regarding AE. Prediction of undetected or emerging hotspots would increase detection rates. Voles and lemmings of the subfamily Arvicolinae are important intermediate hosts in sylvatic transmission systems. Their populations reach high densities in productive grasslands where food and cover are abundant. Habitat availability is thought to affect arvicoline population dynamic patterns and definitive host–intermediate host interactions. Arvicoline habitat correlates with AE prevalence in Western Europe and southern Gansu Province, China.

**Methods and Findings:**

Xiji County, Ningxia Hui Autonomous Region, borders southern Gansu. The aims of this study were to map AE prevalence across Xiji and test arvicoline habitat as a predictor. Land cover was mapped using remotely sensed (Landsat) imagery. Infection status of 3,205 individuals screened in 2002–2003 was related, using generalised additive mixed models, to covariates: gender; farming; ethnicity; dog ownership; water source; and areal cover of mountain pasture and lowland pasture. A Markov random field modelled additional spatial variation and uncertainty. Mountain pasture and lowland pasture were associated with below and above average AE prevalence, respectively.

**Conclusions:**

Low values of the normalised difference vegetation index indicated sub-optimality of lowland pasture for grassland arvicolines. Unlike other known endemic areas, grassland arvicolines probably did not provide the principal reservoir for *Echinococcus multilocularis* in Xiji. This result is consistent with recent small mammal surveys reporting low arvicoline densities and high densities of hamsters, pikas and jerboas, all suitable intermediate hosts for *E. multilocularis*, in reforested lowland pasture. The risk of re-emergence is discussed. We recommend extending monitoring to: southern Haiyuan County, where predicted prevalence was high; southern Xiji County, where prediction uncertainty was high; and monitoring small mammal community dynamics and the infection status of dogs.

## Author Summary

In humans, larvae of the fox tapeworm *Echinococcus multilocularis* typically infect the liver where metastasis, calcification and necrosis cause the zoonotic disease alveolar echinococcosis (AE). Treatment is difficult. Early detection greatly increases patient life expectancy but under-detection is a problem. Understanding the ecological conditions that elevate AE risk would help identify at-risk communities. Voles and lemmings of the subfamily Arvicolinae are important intermediate hosts in most AE endemic areas, and arvicoline habitat has been proposed as a predictor of AE risk. Using a model of spatial autocorrelation with land cover identified from satellite remote sensing imagery, we identified AE hotspots in southern Ningxia Hui Autonomous Region (NHAR), China. Hotspots were not located near optimal arvicoline habitats. Thus, non-arvicolines provide principal reservoirs in NHAR and the range of ecological conditions sustaining *E. multilocularis* transmission in China is greater than previously thought. We also show: social factors explain higher prevalence in females than males; dogs increase infection risk; and we argue that water source quality is important via interaction with other environmental variables. Our map of AE prevalence represents the current state-of-the-art regarding the spatial distribution of AE in southern NHAR and provides an important baseline for future monitoring programs there.

## Introduction

**Figure 1:**
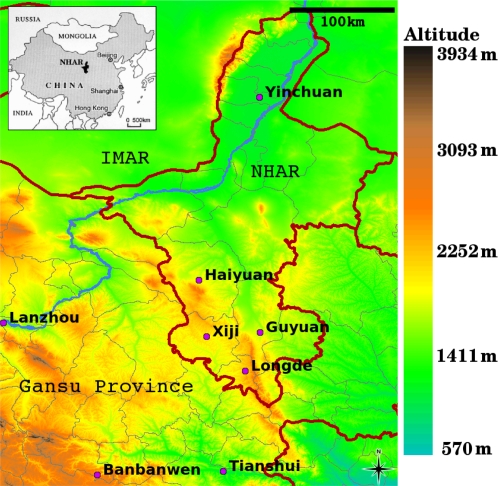
Map of NHAR in relation to Gansu Province and Inner Mongolia Autonomous Region (IMAR) (main) and within China (insert). Provincial boundaries, county boundaries and the Yellow River are marked in red, grey and blue respectively. Principal towns for four counties in southern NHAR are shown. The village Banbanwen, Zhang County, Gansu, where human AE prevalences of 15% have been reported [10], is also marked.

Biological mechanisms known to affect space-time dynamics of infectious diseases include: habitat changes affecting vector breeding sites or reservoir host distributions; niche invasion; biodiversity change including keystone predator loss and rapid magnitudinal increases in reservoir host populations; genetic change in vectors or pathogens; and environmental contamination with infectious agents [1]. Each of these mechanisms may be affected by ecosystem change and the last 50 years have seen the greatest changes in ecosystem structure and function in human history [2]. The Millennium Ecosystems Assessment of the World Health Organisation has listed over thirty infectious diseases known to be affected by ecosystem changes [1]. The list provides compelling evidence that ecological factors affect transmission of many of the most dangerous pathogens and zoonoses. However, the list was incomplete. Its failure to mention the fatal parasitic disease alveolar echinococcosis (AE) reflects that this very dangerous zoonosis is indeed a neglected disease. Despite being globally rare, AE places a serious burden on affected communities in endemic areas and remains very difficult to treat. As for many zoonoses, incidence rates of AE are affected by ecosystem changes. This paper explores the statistical relationships between land cover and human AE prevalence in southern Ningxia Hui Autonomous Region (NHAR) (Fig. 1), China. The identified statistical relationships are then used to map AE prevalence across the area.

Alveolar echinococcosis arises from infection with larvae of the fox tapeworm *Echinococcus multilocularis* [3]. In Europe, prevalences in foxes and the geographical range of the worm have increased giving rise to fears of AE emergence [4],[5],[6]. In central China, high AE prevalences have been reported from: Tibetan pastoral communities of northwest Sichuan [7],[8],[9]; Han communities of southern Gansu [10]; and Hui communities of southern NHAR [11]. A distribution map human AE across China is provided by [12]. The central Chinese endemic area has been described in meta-population terms with the grasslands of northwest Sichuan sustaining a large and stable meta-focus of *E. multilocularis* transmission that feeds peripheral areas where stability is lower by function of reduced availability of, and connectivity between, patches of optimal intermediate host habitat [13]. An average Tibetan pastoralist of northwest Sichuan is estimated to lose 0.81 Disability Adjusted Life Years (DALYs) to alveolar and cystic echinococcosis [14]. Compared to an average 0.18 DALYs lost in the general Chinese population due to all communicable and non-communicable ailments combined [14] it is clear that echinococcosis is a major burden for communities in endemic areas of China and poses a public health problem of primary importance.

Severe liver pathology and metastasis of multilocular cysts during long and asymptomatic incubation periods renders treatment of symptomatic AE cases extremely difficult [3]. Early detection is critical regarding patient life expectancy. Mass screening programs do successfully detect early cases [15],[16], but identifying target areas from hospital records is unreliable in relation to AE [11]. Reliance upon hospital records suffers two problems: i) under-detection in remote areas with limited access to medical facilities or poor knowledge of AE; ii) slow response to epidemiological shifts affected by environmental change. Public health managers would benefit from predictive models that could identify undetected or emergent AE hotspots. Moreover, understanding the links between land cover and small mammal communities is essential regarding development of effective environmentally based disease control strategies.

A classic observation is that voles or lemmings of the subfamily Arvicolinae frequently function as key intermediate hosts for *E. multilocularis* in sylvatic systems [17]. Due to the specific habitat requirements of arvicolines, it has been hypothesised that landscape composition may provide a useful predictor of the spatial distribution of *E. multilocularis* and AE [18],[19]. In eastern France, regular population outbreaks of the vole *Arvicola terrestris* occur in areas abundant with large open patches of pasture and positive correlations between percentage cover of grassland and *E. multilocularis* infection in humans and foxes have been shown [20],[21],[22]. In Zhang County, Gansu, China, trapping frequencies of the vole *Microtus limnophilus* and hamster *Cricetulus longicaudatus* were greatest in grass and shrub patches generated by successional growth following deforestation [23]. Population outbreaks of both species had been reported from the area [24] and species in these genera are highly susceptible to *E. multilocularis* infection [25],[26]. While *C. longicaudatus* was also abundant in lower prevalence agricultural areas, percentage cover of optimal *M. limnophilus* habitat correlated positively with human AE prevalence [10],[12],[27]. In Tibetan pastoral communities of northwest Sichuan modern private fencing practices reduce availability of, and increase grazing pressure on, common lands, improving habitat suitability for *Microtus fuscus* and the lagomorphs *Ochotona curzoniae* and *Ochotona cansus*. Tall grass within fenced areas provides sufficient nutrition and protection to support large populations of arvicolines such as *M. limnophilus* [28]. A correlation between the area of fenced pasture and human AE prevalence has been reported [29]. Details relating to rodent population dynamics and landscape composition are found in [30],[31],[32],[33],[34],[35].

Since economic reform in 1978 China has faced continuous change. The Chinese population has increased from 980 million to 1.3 billion, the urban proportion of which has risen from 20% to 36% and is projected to reach 60% by 2020 [36]. Land cover change in the 1990s encompassed a monolithic 2.99 million hectare increase in cropland and a 0.82 million hectare increase in urban area [36]. In the same period NHAR lost 236700 hectares of grassland and 9200 hectares of unused land and gained 223900, 10100 and 11800 thousand hectares of cropland, woodland and urban land respectively [36]. Proximity to the Gobi desert makes desertification risk in NHAR high [37]. This, and the need for improved flood and erosion management in Yellow River catchments [38], has led to vigorous promotion of afforestation programs [39].

**Figure 2:**
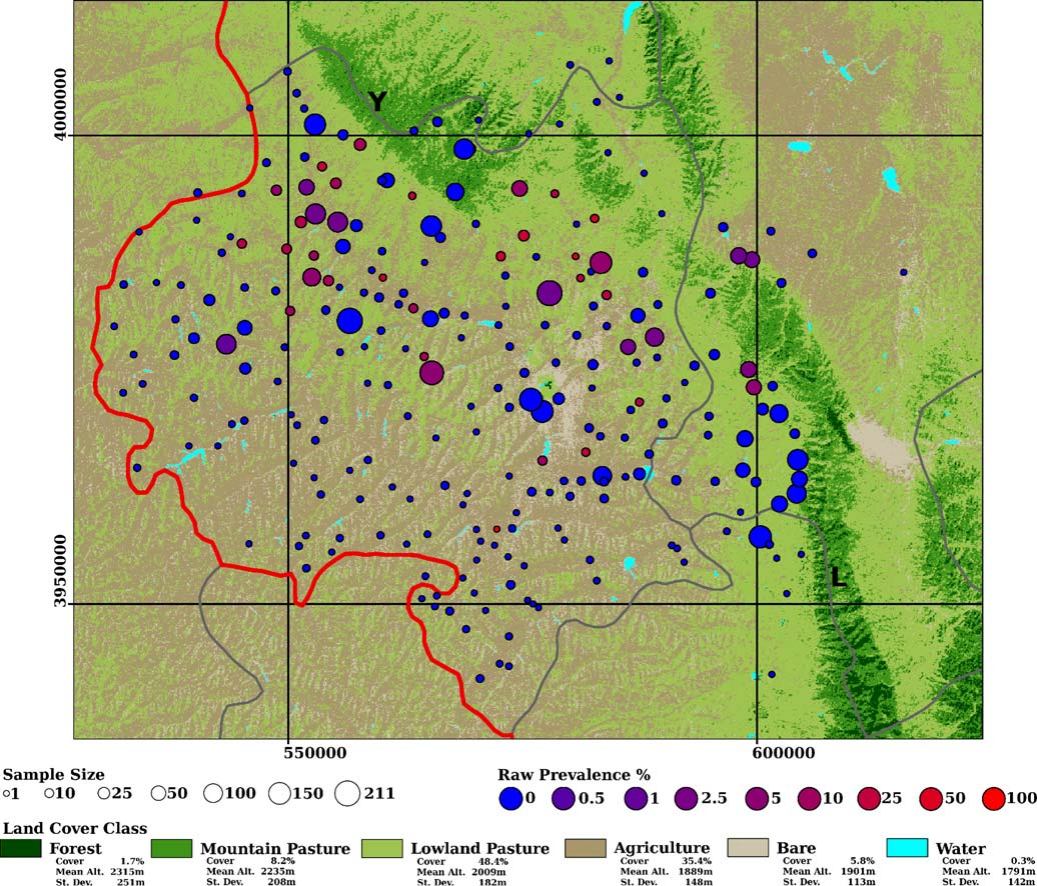
Classification of a 17-4-78 MSS image of Southern NHAR. Mean elevation, its standard deviation and percentage cover of each class were calculated within a rectangular region with upper-left and lower-right coordinates (537690, 4025228) and (625680, 3930016) respectively. The 247 villages within the full dataset are marked, circle size corresponds to sample size and colouration indicates unsmoothed prevalence (100×cases/sample size). Provincial boundaries are marked red and county boundaries are marked grey. The Yueliang range is indicated Y, the Liupan range is indicated with an L. Coordinates are in UTM.

The NHAR is located on the Chinese Loess Plateau. One third of its 5040000 population belongs to the Hui minority [5]. While cystic echinococcosis is found across NHAR, AE has so far only been reported in Xiji, Guyuan and Haiyuan Counties (Fig. 1) [40],[41],[11]. This region lies on the fringes of the known endemic area of central China [42],[12]. The absence of AE north of Haiyuan is attributable to the hot and dry climate [43] providing unsuitable conditions for *E. multilocularis* egg survival [44]. Dominant geographical features of southern NHAR include: the Liupan mountain range (2927 m); the Yueliang mountain range (2626 m); and the Nanhua mountains (2941 m) just south of Haiyuan City (Figs. 1 & 2). Mountain vegetation is dominantly lush grassland, although some residual forest patches persist in the southern Liupan. Elsewhere land cover is dominated by agricultural fields with hill tops reserved for pasture, albeit of much lower quality than in the mountains.

The current work investigates the hypothesis that AE in Xiji County is attributable to its mountain areas where lush grasslands provide optimal habitat for large, possibly cyclical, arvicoline populations, and relatively temperate climates favour *E. multilocularis* egg survival. This extrapolation of results from Zhang County [10],[12],[27] assumes that the endemic transmission of the two areas functions within comparable sets of environmental conditions. Specifically, the landscape - disease correlates observed in Zhang are tested as predictors of AE prevalence and maps of predicted prevalence and associated uncertainties are presented.

## Materials and Methods

Records from Xiji (35°33′N - 36°13′N×105°18′E - 106°04E) County Hospital dating from 1985 motivated a mass screening program in 2002–03 described in [11]. Stations for performing abdominal ultrasound screening were established at medical centers or schools in 26 villages. Many people from surrounding villages also participated and in total 4778 individuals in the age range 5–83 years were screened. Approval for the surveys was given by the Ethics Committee of Ningxia Medical College, and written consent was obtained from all adult participants and from parents of minors aged 5 years or older who agreed to participate. Personal details, responses to a knowledge, attitudes and practices questionnaire and AE status were recorded in EpiInfo [45]. These records were combined with spatial coordinates for each village enabling storage in GRASS GIS [46] and spatial analysis in R [47]. The current study used a subset of this data corresponding to those 247 villages which could be geolocated within the study area. Since no AE cases were observed among the 1426 participating students analyses were further restricted to the non-student subset of data. This sub-sample, consisting of 3205 individuals from 152 villages, is summarised in Table 1.

**Table 1:** Prevalence for subsets of the current dataset with 95 percent confidence intervals.

Subset	*c*/*n*	Prev %	Subset	*c*/*n*	Prev %	χ^2^	*p*
Full Data	96/3205	3.00	-	-	-	-	-
		(2.45–3.66)					
Female	60/1681	3.57	Male	36/1524	2.36	3.6	0.057
		(2.76–4.6)			(1.68–3.29)		
Hui	60/1567	3.83	Han	36/1638	2.20	6.78	0.0092
		(2.96–4.93)			(1.57–3.06)		
Farmer	91/2999	3.03	Non-Farmer	5/206	2.43	0.0802	0.777
		(2.46–3.73)			(0.897–5.88)		
Tap or Well	47/2197	2.14	Other Water Sources	32/846	3.78	5.89	0.0152
		(1.59–2.86)			(2.64–5.36)		
Owned Dogs	34/875	3.89	Haven't Owned Dogs	44/2253	1.95	8.9	0.00285
		(2.75–5.45)			(1.44–2.64)		

Comparable subset pairs are compared by the χ^2^ test with one degree of freedom. *c*/*n* indicates the number of AE cases *c* in a subsample of size *n*.

Since latency of AE in humans is long, hepatic lesions detected during mass-screening likely arose from infection events occurring ten years or more prior to screening. In order to analyse the effects of land cover on AE distribution it was pertinent to work with archived satellite remote sensing data. For this a Landsat Multi-Spectral Scanner (MSS) image (acquisition date, April 1978) was used. Five ground control points (GCPs) were collected across the study area by hand-held global positioning system (GPS) for the purpose of geocorrection. These GCPs, typically road junctions or river bridges, were unidentifiable in the 60 m resolution MSS image. Therefore, a Landsat Enhanced Thematic Mapper (ETM) image with a 15 m panchromatic layer (acquisition date, June 2001) was obtained. The GCPs indicated that the pre-purchase automated geocorrection of the ETM image was subject to a georectification error of 250 m in the north west of the study area. A fist order polynomial geocorrection model with nearest neighbour resampling was used to reduce this error (RMSE = 11 m). The MSS image was then georectified to the corrected ETM using larger features identifiable in both images.

Land cover was assessed via a photographic survey in July 2002. Over 250 photographs of the landscape were taken across Xiji, Guyuan and Haiyuan counties. The point from which each photo was taken was recorded using a hand-held GPS receiver and the orientation of the camera was measured with a hand-held compass. These data enabled identification of over 140 homogeneous patches within the ETM image. Training area identification for classification of the MSS image required collection of historical information. This was achieved by discussing land cover change history with local farmers and pastoralists. Training areas were then identified on the basis of these local reports, field data from 2002 and image analysis. A land cover map (Fig. 2) was derived from the MSS image using supervised maximum likelihood classification [48] with the following classes: water bodies; forest; agricultural fields; bare soils; mountain grasslands and lowland pasture. A qualitative assessment of the classified image indicated good correspondence with the available information on historical land cover. All image processing was performed using Erdas Imagine 8.4 [49]. Under the hypothesis, mountain grasslands were expected to have provided both optimal key reservoir habitat and optimal climatic conditions regarding egg longevity. Therefore, that cover class was expected to be positively associated with areas of greatest human AE risk.

Areal cover for each land cover class was estimated as the proportion of pixels belonging to the class in question within a circular buffer centred at a given pixel. This was repeated for every pixel in the image, a technique sometimes referred to as moving window analysis. Such metrics vary with respect not only to landscape composition but also to the buffer radius *R*. It is generally impossible to anticipate *a priori* a suitable value for *R* so a set of values, *R*∈{500*m*,1000*m*,2000*m*,3000*m*,…,20000*m*}, was considered. The most pertinent value for *R* was identified via Akaike Information Criterion (AIC) based model selection (described below).

Generalised additive models (GAMs) with a logistic link function [50] were used to investigate correlations between risk factors and AE status (presence/absence of hepatic AE) of subjects. The model included the factors: gender (female), ethnic group (Hui), occupation (farmer), dog ownership and water source (tap or well). Non-linearity between age and the logit of prevalence was modelled using a cubic regression spline as described in [50]. Landscape effects were then investigated by adding areal cover estimates of mountain pasture and lowland pasture to the model as linear effects. The optimal *R* for each class was estimated by an exhaustive comparison of AIC among all possible models. This procedure was also repeated with areal cover estimates of forest included in the model.

The model outlined above was then analysed in a Bayesian context using the software BayesX [51]. This enabled two additional sources of variation to be investigated in a generalised additive mixed model (GAMM) approach: within-village random effects to account for village specific peculiarities in prevalence arising from unobserved village specific factors; and a spatial random effect to account for additional spatial autocorrelation. The spatial random effect was modelled as a Markov random field [52],[53] on a 53×44 grid with a 2 km×2 km pixel resolution. Non-linearity in age was modelled using a degree 3 P-spline, with second order random walk penalty against over fitting, on 20 equidistant knots [54]. Non-informative inverse gamma priors were assumed for variance components of the P-spline and random effects with hyper-parameters a = 0.001 and b = 0.001. Models with no random effects, village random effects only, spatial random effects only and both village and spatial random effects were compared with and without pasture and forest areal cover estimates. Buffer radii for the three land cover classes were fixed at the optimal values identified above. The parameter space of the Bayesian GAMMs was sampled using Markov Chain Monte Carlo (MCMC) techniques. For each model, MCMC was run for 1200000 iterations, discarding an initial burn-in period of 100000 iterations and sub-sampling every 10th sample thereafter. Model comparison was performed using the deviance information criterion (DIC) [55] with model selection based upon the model returning the lowest DIC.

A map of AE prevalence, corresponding to the age group for which the expected value of the P-spline was zero, was derived from posterior means of all other model parameters (except age), population means for individual-level risk factors and the areal cover layers. To avoid visual artifacts arising from the resolution differences of the land-cover data and the MRF, simple kriging [56] was used to interpolate MRF posterior means, evaluated at all pixel centroids and sampled villages, to a 60 m resolution grid. This interpolation used a linear semi-variogram model without a nugget and a fixed range of 60 km. Uncertainty in the spatial random effect was visualised by mapping the range of the 95% credibility interval of the MCMC samples of the MRF. As a model check, standardised village residuals were calculated according to [57] and a semi-variogram [58] was used to check for the absence of systematic spatial variation in model residuals. Geostatistical analyses were performed using the R implementation of the gstat package (http://www.gstat.org/s.html). Maps were plotted using the R function spplot.

## Results

Prevalence of alveolar echinococcosis in the studentless subset of data was 3.0% (2.45–3.66, 95% C.I.) (Table 1). Reported occupations among the 96 AE positive subjects were: 91 farmers; three housewives (aged 42,46 and 50); one cadre (aged 50); and one worker (aged 33). There were five cases in the under 30 age group (15, 18, 23, 23 and 25 years). Univariate analysis of the studentless sub-sample detected no significant difference in AE prevalence between farmers and non-farmers (χ^2^ = 0.0802, p = 0.777). Significantly higher AE prevalence was detected among Hui than among Han (χ^2^ = 6.78, p = 0.0092). AE prevalence was lower among subjects with access to tap or well water (χ^2^ = 5.89, p = 0.0152). Evidence of a sex difference in prevalence was weaker in the studentless subset (χ^2^ = 3.6, p = 0.057) than in the unfiltered subset (χ^2^ = 8.15, p = 0.0043). There was a sex bias in the student population with a greater number of students being male than expected under an equality null hypothesis (χ^2^ = 96.8, p<2.2×10^−16^). There was a sex bias in the farming population with a greater number of farmers being female than expected under equality conditions (χ^2^ = 115.4264, p<2.2×10^−16^). Dog ownership was reported more frequently among Han than Hui (χ^2^ = 107.3, p<2.2×10^−16^).

In interviews in 2002 local farmers reported that during the late 1970s valleys and lower slopes were generally used for agricultural crop production while upper slopes and hill tops were reserved for grazing. At that time there were no livestock restrictions. Twenty to fifty sheep per family was not uncommon and grazing pressure had been intense. Farmers also reported that the last patches of “forest” had been located on hill tops. However, these “forests”, by comparison with known forest patches in the southern Liupan, were not evident in the MSS imagery suggesting either small patch size or low tree density. Farmers reported that the number of sheep per household was capped in the late 1990's when incentives for converting grazing land to tree or shrub plantations were put in place.

The classified image is presented, with some summary statistics on percentage cover in Fig. 2. Two classes dominated the classification: agriculture and lowland pasture. The former was dominant in valleys and on hillsides while the latter was dominant on hill tops and in areas fringing the larger mountains. At higher elevations the Liupan, Yueliang and Nanhua mountains were dominated by mountain pasture and, particularly in the southern Liupan, harboured almost all the remaining forest in the area. Given the absence of archived land cover data, quantitative accuracy assessment was not possible. However, the classified image corresponded well with both field observations made in 2002 and anecdotal reports of historical land cover provided by local pastoralists and farmers. The mean normalised difference vegetation index, calculated from a Landsat MSS image acquired on June 1975, was 0.34 (s.d. = 0.13) and 0.14 (s.d. = 0.09) in mountain and lowland pastures respectively (*t* = 536.0, df = 152101.4, p<2.2×10^−16^).

The buffer radii maximising the likelihood of the GAM were found to be 6 km, 15 km and 18 km for lowland pasture, mountain pasture and forest cover respectively. Omitting forest cover resulted in optimal radii of 7 km and 15 km for lowland pasture and mountain pasture classes respectively. Models including the spatial random effect consistently returned lower DICs than their non-spatial counterparts (Table 2). The lowest DIC was returned by the model with all three areal cover components. However, this model was not selected for inference or prediction because of fears that the spatial relation between forest and sampling points was causing over fitting on a small subset of the data, namely the villages of the Liupan mountains in Guyuan and Longde counties closest to the largest forest patches and in which few AE cases were observed (Fig. 2). Village-level random effects failed to reduce the DIC of models containing a spatial random effect. Therefore model 2 in Table 2 was chosen for further inference and prediction.

**Table 2:** Model comparison with deviance information criterion (DIC).

Model	*LP*	*MP*	*F*	*spatial*	*village*	DIC
1	•	•	•	•		554.573
2	•	•		•		555.596
3	•	•		•	•	555.849
4	•	•	•	•	•	556.151
5				•		556.982
6	•	•	•		•	562.781
7	•	•			•	566.430
8					•	567.348
9	•	•	•			601.904
10	•	•				624.773
11						654.245

All models bellow included gender, ethnic group, farming, dog ownership and tap or well as a water source as fixed effects plus a P-spline term on age. All other models were nested on this model and additional terms are indicated with a •. Spatial and village level random effects are depicted by *spatial* and *village* respectively. Areal cover of lowland pasture, mountain pasture and forest classes are depicted by *LP*, *MP* and *F* respectively.

**Figure 3:**
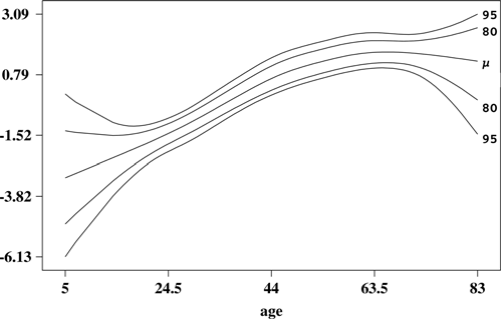
Posterior mean with 80% and 95% credibility intervals for the effect of age on the logit of prevalence.

Non-linear age-specific adjustments to the logit of prevalence are represented by the posterior mean and the 80% and 95% credibility intervals of the P-spline in Fig. 3. The fitted spline indicates a linear augmentation of prevalence on the logit scale in the 5–50 years age range, although uncertainty in the 5–20 years range was large. The linear trend plateaus at about 60 years. The expected value of the spline was closest to zero for 38 year olds. i.e. prevalence in that age category was representative of the average situation while prevalences in younger and older age groups were lower and higher than average respectively. Moreover, prevalence at 38 years could be predicted simply without adjustment for the non-linear age effect.

The mean, standard deviation, median and 95% credibility intervals of posterior samples for regression coefficients are shown in Table 3. The ranges of the 95% credibility intervals of posterior samples were strictly positive for dog ownership and lowland pasture and strictly negative for the intercept and mountain pasture. The 95% credibility intervals of coefficients of all other factors were not exclusive of zero. Posterior means and standard deviations of variance parameters for the P-spline and Markov random field were 0.0675 (s.d. 0.138) and 4.89 (s.d. 2.21) respectively.

**Table 3:** Summary statistics of posterior samples for regression coefficients of model 2 (see Table 2).

Variable	Mean	StDev	2.5%-Quant	Median	97.5% Quant	
Intercept	−7.97	1.56	−11.2	−7.92	−6.00	•
Female	0.385	0.265	−0.129	0.383	0.726	
Hui	0.480	0.475	−0.474	0.486	1.0814	
Own Dog	0.602	0.296	0.0190	0.602	0.981	•
Farmer	0.253	0.634	−0.900	0.222	1.0777	
Tap or Well	−0.0469	0.306	−0.639	−0.0498	0.346	
Lowland Pasture	0.000207	9.20e-05	2.84e-05	0.000206	0.000324	•
Mountain Pasture	−0.000118	4.81e-05	−0.000218	−0.000116	−5.89e-05	•

The final column indicates whether zero lies outside the 95% credibility interval.

**Figure 4:**
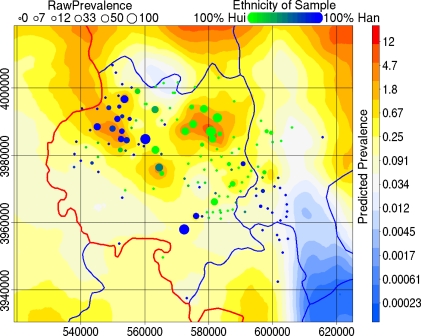
Predictions of human AE prevalence from a Bayesian GAMM of trends in human AE prevalence combined. Trend terms were mountain pasture (dark blue areas) and lowland pasture (yellow areas). The model succeeds in smoothing the raw prevalence (100×cases/sample size) to within a plausible range. A hot-spot is identified in the area between the Liupan Shan and Yueliang Shan. The provincial boundary is marked black, county boundaries are marked blue. A non-random spatial distribution of the two major ethnic groups in the area is evident.

**Figure 5:**
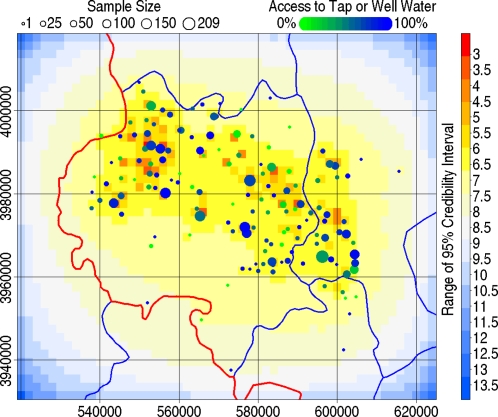
The range of the 95% credibility interval from the posterior simulations of the spatial random effect. Sample sizes at each village are indicated by circle size. The proportion of the sample at each village with access to tap or well water is indicated by colour.

Figure 4 presents the predicted prevalence among 38 year olds. The range of the 95% credibility intervals for each pixel of the spatial random effect is presented, with sample size, in Fig. 5. The principal hotspot was nested between the Liupan and Yueliang mountain ranges and lies within an area where the population was dominantly Hui (Fig. 4). Two lesser hotspots were also evident: the second, approximately 30 km west of the first, was situated in a dominantly Han area; and the third, situated between and a little south of the first and second, was in an ethnically mixed area. The lowest predicted prevalences were associated with the Liupan and Yueliang mountains (Fig. 4) despite relatively intense sampling in southwest Guyuan County (Fig. 5).

## Discussion

No AE cases were detected in students. Coefficient estimation for that subpopulation was neither necessary nor feasible, thus students were removed from further analyses. The remaining subsample consisted largely of farmers and no further statistical difference in AE prevalence between farmers and non-farmers was detected. Higher AE prevalence in Hui than in Han and among those without access to tap or well water was observed by univariate but not by multivariate statistics. These discrepancies probably arose from the non-random spatial distribution of the two ethnic groups (Fig. 4) and water source quality within the study area (Fig. 5). The spatial random effect apparently nullified the water source and ethnicity effects suggesting spatial heterogeneity in effect size. It is likely that a larger proportion of Hui than Han were infected by function of the spatial distribution of the two ethnic groups relative to areas ecologically favourable (by which we include water source quality) to *E. multilocularis* transmission. Redundancy of ethnicity in a spatial model and the fact that the high endemicity area in Zhang and the secondary hotspot here were both Han areas negates possible arguments of large between group genetic differences in susceptibility. Spatial heterogeneity in effect size could arise naturally for ecological reasons, e.g. interaction with spatially heterogeneous variables such as transmission intensity or density of *E. multilocularis* eggs in the environment. The role of unmeasured socio-economic factors in this interaction is impossible to assess here.

The only putative risk factor to correlate with AE prevalence in the Bayesian analysis was dog ownership. This corroborates previous studies suggesting that domestic dogs play an important role in the epidemiology of AE. Higher AE prevalence among females is frequently reported [59]. Both accelerated growth of *Echinococcus* cysts in immunosuppressed pregnant women [60],[61] and risk behaviour frequency (dog contact, farming,…) [10],[11] have been suggested as explanations, but the relative contributions of biological and sociological factors are unclear. Here, a sex difference was observed in the unfiltered dataset, but evidence for the sex difference became weak after students were removed from the analysis. The observed sex biases in the student and farming populations sufficiently explain the observed sex difference in AE prevalence.

Classical prevalence estimates obtained from small samples typically suffer low precision and are misleading. The village Xiping (UTM 572244, 3957984) gives a perfect example, a sample of just one individual provided a classical prevalence estimate of 100% (Figs. 4 and 5). One of the greatest strengths of spatial statistics over classical statistics is that spatial models utilise neighbouring information to tighten confidence intervals at each point. A good spatial model smooths out spuriously large variation arising from sampling without over-smoothing the true variation in the underlying phenomena being studied. The predicted prevalence surface presented here clearly achieves this goal, village-level prevalence estimates were restricted from the unrealistic range 0%–100% to a range comparable to other areas within the central Chinese endemic area (Fig. 4) [10],[29]. Over-smoothing appears to have been avoided given the size of the identified clusters relative to the spatial distribution of sampled villages. Estimated prevalence cannot be properly assessed without reference to a measure of uncertainty. There is a clear inverse relation between local sampling density and uncertainty (Fig. 5). Areas north and west of the Yueliang and near the Longde County - Gansu border are predicted by extrapolation of landscape indices to have high prevalence (Fig. 4). There is currently no data from these areas and follow up studies would help assess the validity of these extrapolations where uncertainties are large.

A hotspot located between the Liupan and Yueliang mountains was identified. This hotspot includes Nanwan village (UTM 583340, 3986433) where a large familial cluster of AE cases has been described [62]. Here we show that clustering has occurred at a higher organisational level than the family. The hotspot was approximately 10–15 km in diameter (Fig. 4). Further research is required to identify the causative factors of this hotspot. A direct effect of climate is an unlikely explanation since the hotspot is not within the mountains where *E. multilocularis* egg survival would be greatest. The relative roles of fox, dog and intermediate host densities and interactions with socio-economic factors must be identified. In a previous study, a family in which four in eight members were infected reported hunting *Spermophilus* for food and feeding uncooked viscera to their dogs [59]. Future studies must assess whether this practice was: unique to this family; common within the hotspot; or was wide spread across Xiji County.

The landscape analysis suggests that the environmental conditions favouring *E. multilocularis* transmission in Xiji differ from those favouring transmission in southern Gansu. Areal cover of mountain pasture around villages did correlate to AE prevalence, but with a negative coefficient. By contrast, abundance of the more degraded lowland pasture was associated with higher human AE prevalence. Note that these observed patterns depend upon the choices of scale that define the current sampling design. A hypothetical extension of the study area by an order of magnitude might result in contrary findings regarding the role of the Liupan mountain range. The greatest difficulty in interpreting this result arises from the time lags, which are unavoidable given the slow development of AE in humans, inherent in the current study. The lack of archived ground data makes it is hard to know exactly what constituted vegetation cover in “lowland pasture” areas. The available evidence comes from field observations (2001–03), image analysis of archived remote sensing data (1975 & 1978) and anecdotal accounts of local farmers. On this basis, “lowland pasture” most likely represents areas of grass, heavily grazed by sheep and goats and possibly interspersed with sub-pixel remnants of forest or shrub cover. Despite this uncertainty, one result is clear, the hypothesis of the current study fails to describe the Xiji endemic zone and small mammals of the Liupan grasslands were not the principal reservoirs of *E. multilocularis* linked to AE infection in humans. So were the principal intermediate host reservoirs in Xiji County different to those of Zhang where *Microtus limnophilus* was central to the eco-epidemiology of human AE?

A small mammal survey in 2003 recorded 16 species belonging to 7 families and identified five small mammal assemblages [63]. Forest, shrub and grasslands located in mountains provided greater diversity and lower trapping frequencies than lower elevation habitats. *Microtus fortis* and the wood-mouse *Apodemus peninsulae* were only trapped in forest and dense shrub where they dominated trapping results. Mountain grasslands, dominated by the lagomorph *Ochotona huangensis*, provided the highest densities of *Apodemus agrarius* and the jumping mouse *Eozapus setchuanus* was trapped uniquely there. Diversity was lowest and trapping frequency greatest in newly aforested set-aside where hamsters (*Cricetulus longicaudatis*) were dominant. This habitat provided the greatest trapping frequencies for *Mus musculus*, *Ochotona daurica* and the semi-desert jerboas *Dipus sagitta* and *Allactaga sibirica*. These species were also present in ploughed fields where the hamsters *C. longicaudatus* and *Tscherskia triton* and the zokor *Eyospalax fronteria* (previously *Myospalax fontanierii*), all known agricultural pests, were dominant. *Dipus sagitta*, *A. sibirica* and *O. daurica* were not trapped in areas of more advanced afforestation where hamsters and the Sciuridae *Spermophilus alashanicus* were dominant. Importantly, Arvicolidae were not trapped in large numbers in any habitat [63].

Susceptibility to *E. multilocularis* is undocumented for many of these species so inference must be made at higher levels biological organisation. Cyst fertility is poor in *Apodemus* [64] and *Spermophilus* [17],[65]. Fertile cysts in nine *Eyospalax fronteria* from the area contained few protoscoleces [40] and in Zhang *E. fronteria* was more abundant in lower prevalence agricultural areas [23],[27]. *Allactaga elater* has been found naturally infected in Azerbaidzhan [32]. The gerbil *Meriones unguiculatus* which has similar habitat preferences to jerboas has been reported in the area [40]. Gerbils [66],[67] and hamsters [26] provide excellent laboratory models for *E. multilocularis* and natural infections have been found in *M. unguiculatus* in NHAR [17] and in *C. kamensis* in Sichuan [unpub data]. *Ochotona daurica* has been found infected in Tuva, southern central Russia [17] and *O. curzoniae* is frequently present in fox faeces from NW Sichuan where it is predated preferentially, even when hamsters are visibly abundant [unpub data].

Each habitat in Xiji hosts potential *E. multilocularis* intermediate hosts. Foxes predate preferentially when a preferred prey species becomes readily available so high densities of preferred susceptible prey provide optimal conditions for *E. multilocularis* transmission [19]. Low densities and high diversity of small mammals in mountain habitats may explain the negative correlation between this habitat and AE prevalence. By contrast AE prevalence is rarely high in large agricultural expanses despite high densities of hamsters and zokors, suggesting low levels of predator prey interaction in these landscapes of intense human activity. In Xiji, AE risk was positively correlated with habitat best described as non-montane, non-agricultural with abundant bare soil, thin shrub cover and probably supporting large densities of hamsters, jerboas, *O. daurica*, zokors and mice, all of which may contribute to transmission.

The current work indicates that *E. multilocularis* can sustain transmission through small mammal communities that are not dominated by large cyclic populations of arvicolines. The meta-population dynamics of *E. multilocularis* across central China functions through a diversity of intermediate host communities. This diversity is largely unknown, the most up-to-date reference being [68], an atlas of the sylvatic mammals of China. But low spatial precision and a lack of information regarding dynamic patterns compromises its utility for modelling. It seems species level population data spanning large areas of China is not available, although such a dataset would be invaluable regarding the ecological management of small mammals and their related diseases.

During the 1990's rodenticides were applied liberally across much of central northern China [69]. Secondary poisoning destroyed dog populations in both Xiji and Zhang [11],[62],[13]. Many predator species disappeared [69] which would have inadvertently reduced transmission and may explain the absence of AE in children aged less than 15 years. Rodenticides are now heavily controlled and the domestic dog population is growing again, in part, courtesy of illegal dog trafficking with dogs from Tibetan areas being particularly appreciated. So the scene is set for a re-emergence of AE in Xiji and serology studies in school children have detected anti-*E. multilocularis* antibodies suggesting transmission is currently active in the area [70].

To conclude, landscape analyses indicate that mountain grasslands correlated negatively to AE prevalence in Xiji. This suggests transmission of *E. multilocularis* in Xiji, somewhat uniquely, functions principally through non-arvicoline species. The meta-population dynamics of *E. multilocularis* in central China functions across a diversity of eco-zones and small mammal communities which remain largely unknown. One principal and two lesser hotspots, each approximately 15 km in diameter, were identified. This spatial clustering appears to be caused by complex interactions between social and environmental factors that warrant further study. Spatial heterogeneity in water source quality apparently contributed, via interaction with other factors, to the observed distribution of AE. Sex biases in the student and farming populations appears to explain higher AE prevalence in females and dog ownership was a positive risk factor. Extrapolation of landscape trend terms identified three areas of above average prevalence, west of the southern Liupan and west and north of the Yueliang mountains, although uncertainty was large beyond the corpus of the sampling design. The prevalence and uncertainty maps represent the current state of the art regarding what is known of AE distribution in Xiji and provide an important baseline for future epidemiological monitoring and eco-epidemiological investigation. Future mass screening could focus on villages with a history of low quality water supply and should extend the study to: southern Haiyuan County where high prevalence is predicted; and southern Xiji County where uncertainties were large and a small number of cases have been detected despite low sample sizes. Extending the study area would also extend the range of environmental variation covered within the dataset which would be important regarding further data mining for ecological covariates and interactions.
